# Comprehensive insights into the function and molecular and pharmacological regulation of neuron-derived orphan receptor 1, an orphan receptor

**DOI:** 10.3389/fphar.2022.981490

**Published:** 2022-08-30

**Authors:** Hongxiang Hong, Jianbin Su, Chao Huang, Xu Lu, Zhiming Cui

**Affiliations:** ^1^ Department of Spine Surgery, The Second Affiliated Hospital of Nantong University, Nantong, China; ^2^ Department of Endocrinology, The Second Affiliated Hospital of Nantong University, Nantong, China; ^3^ Department of Pharmacology, School of Pharmacy, Nantong University, Nantong, China

**Keywords:** Nor1, function, regulation, orphan receptor, pharmacology

## Abstract

Neuron-derived orphan receptor 1 (NOR1), also called nuclear receptor subfamily 4 group A member 3 (NR4A3), is a nuclear receptor belonging to the NR4A family. Since no endogenous ligand has been identified to date, NOR1 is also referred to as an orphan receptor. NOR1 is expressed in a variety of cells and tissues, including neurons, vascular smooth muscle cells, T lymphocytes, dendritic cells, tumor cells, heart, liver, and pancreas. Because NOR1 was first identified in apoptotic neurons, it is functionally associated with the regulation of cell migration and the growth of neuronal synapses. In-depth studies have shown that NOR1 can be edited by the immediate early gene and functions as a transcription factor. NOR1 has been shown to be rapidly induced by a number of stimulants including growth factors, fatty acids, and neurotransmitters. Elevated NOR1 levels may be involved in a number of pathophysiological processes. These include regulation of cellular apoptosis and regeneration, neuron formation, contextual fearing memory, inflammation, vascular smooth muscle proliferation, insulin secretion, and tumor development, whereby NOR1 mediates the pathogenesis of numerous diseases such as cerebral ischemia, depression, post-traumatic stress disorder, atherosclerosis, abdominal aortic aneurysm, cardiac hypertrophy, diabetes, osteoarthritis, rheumatoid arthritis, and cancer. However, to date, comprehensive insights into the function of NOR1 are not available in sources published online. In this review, we provide a brief overview of the function and molecular and pharmacological regulation of NOR1 in various pathological or physiological conditions to advance the development of NOR1 as a novel target for disease treatment.

## Introduction

Neuron-derived orphan receptor 1 (NOR1) is a nuclear receptor that belongs to the NR4A family, which also includes Nur77 (also called nerve growth factor-induced gene B (NGFI-B) or NR4A1) and the closely related proteins Nurr1 (also called NR4A2) and NOR1 (also called nuclear receptor subfamily 4 group A member 3 (NR4A3)) (Ranhotra, 2015; Chen H. et al., 2020). It is expressed in a variety of cells and tissues, including neurons (Chio et al., 2016), vascular smooth muscle cells (VSMCs) (Takahashi et al., 2019), T lymphocytes (He, 2002), dendritic cells (Nagaoka et al., 2017), tumor cells (Cai et al., 2020), heart (Cañes et al., 2020), liver (Pei et al., 2006), and pancreas (Close et al., 2019). Structural studies have shown that NOR1 has a conserved molecular structure consisting mainly of a non-conserved N-terminus with a ligand-independent activation function-1 (AF-1) domain, a highly conserved DNA-binding domain (DBD), and a moderately conserved C-terminus with a ligand-binding domain (LBD) and a ligand-dependent AF-2 domain (Giguère, 1999). The DBD, which exhibits a high degree of sequence homology among the three NR4A family members, contains a nuclear localization sequence that determines the distribution of NOR1 in the nucleus (Giguère, 1999). Similar to the other members, the endogenous ligand of NOR1 is unclear and the transcriptional activity of NOR1 is independent of the binding of an endogenous ligand (Martínez-González et al., 2021), suggesting that NOR1 is constitutively active. Moreover, similar to the other members, NOR1 binds as a monomer to the promoters of AAAGGTCA target genes containing the response element of NGFI-B, or as a homodimer or heterodimer to recognize a Nur-Response Element consisting of two inverted NBREs separated by five nucleotides (GGT​TCA​CCG​AAA​GGT​CA) (direct repeat 5, DR-5) (Perlmann and Jansson, 1995; Zetterström et al., 1996; Martínez-González and Badimon, 2005; Evans and Mangelsdorf, 2014). However, despite the above similarities, NOR1 sometimes differs from Nur77 and Nurr1. For example, researchers have found that Nur77 and Nurr1, but not NOR1, heterodimerize with the retinoid X receptor (RXR) (Martínez-González et al., 2021) and NOR1 generally has a lower ability to activate gene transcription due to its low affinity for this response element (Maira et al., 1999). In-depth studies have shown that NOR1 expression and function can be regulated by a variety of factors, including peptide hormones, growth factors, cytokines, and cellular stressors (Williams and Lau, 1993; Deutsch et al., 2012; Safe et al., 2014). Functional NOR1 has been reported to be involved in the regulation of a variety of biological processes, including metabolism (Ranhotra, 2015; Crean and Murphy, 2021), neuronal survival (Chio et al., 2016), vascular homeostasis (Rodríguez-Calvo et al., 2013; Takahashi et al., 2019; Martínez-González et al., 2021), inflammatory responses (Calvayrac et al., 2015; Qing et al., 2017), and carcinogenesis (Yi et al., 2017; Cai et al., 2020). In the present review, we summarized the current knowledge on the function of NOR1 based on the previously published literature, and discussed the factors or drugs that regulate NOR1 expression and function. Our work may pave the way to advance the development of NOR1 as a new target for disease treatment.

### The role of NOR1 in the nervous system

**FIGURE 1:**
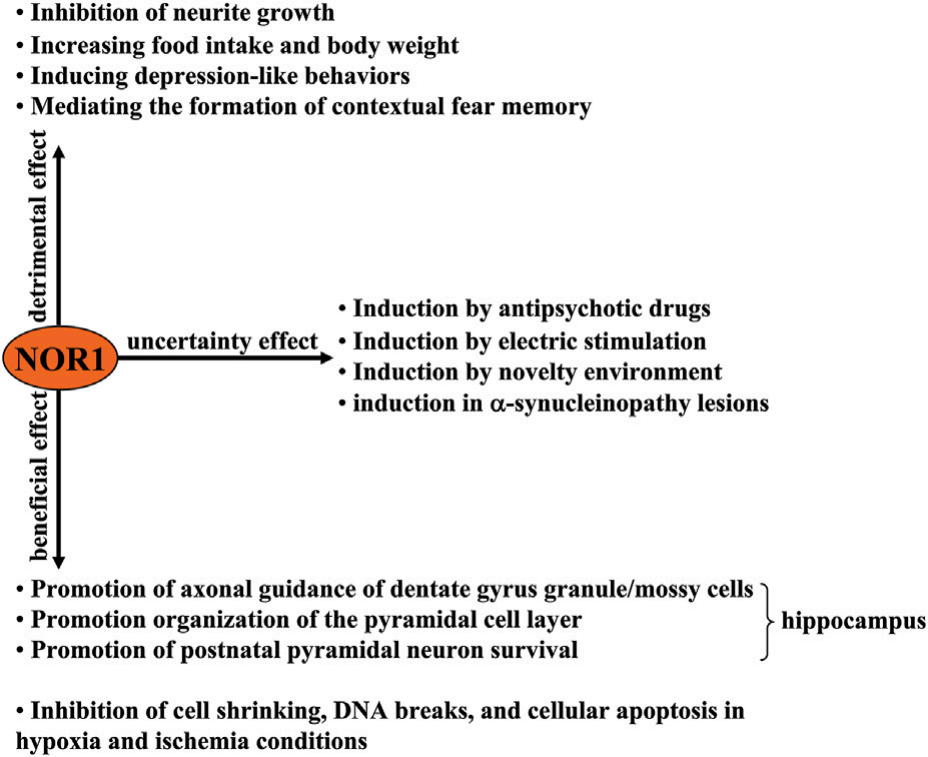
A schematic representation of the beneficial, harmful, and uncertain effects of NOR1 in the nervous system. Beneficial effects of NOR1 include inhibition of cell shrinkage, DNA breaks, and cellular apoptosis under hypoxia and ischemia conditions, as well as promotion of axonal guidance of dentate gyrus granule/mossy cells, organization of the pyramidal cell layer, and survival of postnatal pyramidal neurons. The deleterious effects of NOR1 include inhibiting neurite growth, increasing food intake and body weight, inducing depression-like behaviors, and mediating the formation of a contextual fear memory. The functional significance of its induction by antipsychotic drugs, electrical stimulation, novelty environment, and α-synucleinopathy lesions remains unclear.

In the nervous system, NOR1 was first identified in rat apoptotic neurons (Ohkura et al., 1996). Its cDNA encodes a protein that has a molecular weight of 68 kDa and 628 amino acid residues (Ohkura et al., 1994). Genetic analysis had shown that NOR1 mRNA is highly expressed in the forebrain of fetal mice on day 17 of the embryonic period and is downregulated to low levels in several regions of the adult brain, including the hippocampus, striatum, nucleus accumbens, and prefrontal cortex (Zetterström et al., 1996). The functions of NOR1 in the nervous system are still largely unknown. Here, we discussed the published evidence to date on the role of NOR1 in the nervous system, including its beneficial, harmful, and uncertain aspects (Figure 1).

### Beneficial effect of NOR1 in the nervous system

Some studies had reported that NOR1 plays a crucial role in neuron survival. The first evidence for this opinion came from a NOR1 ablation experiment in mice, in which a vector carrying a 10-kb fragment of the mouse NOR1 gene interrupted within exon 2 by the introduction of a β-galactosidase reporter gene (lacZ) and a neomycin selection cassette downstream of the ATG translation initiation codon and upstream of the DNA-binding domain of NOR1, was inserted into the genomic locus of the NOR1 gene (Ponnio et al., 2002). This targeted integration, which disrupts the protein at amino acids 212–231 (Ponnio et al., 2002), was found to cause abnormal axonal guidance of the dentate gyrus granule and mossy cells, disorganization of the pyramidal cell layer, as well as early postnatal death of pyramidal cells in the CA1 region of the hippocampus, which subsequently increases susceptibility to the excitotoxic glutamate receptor agonist kainic acid (Pönniö and Conneely, 2004), suggesting that physiological NOR1 helps postnatal neurons reach an appropriate functional state. In an *in vitro* hypoxia model, oxygen/glucose deprivation in cultured Neuro-2a cells was reported to increase NOR1 expression levels in a cyclic adenosine monophosphate (cAMP) response binding protein (CREB)-dependent manner, which as associated with cell shrinkage, DNA breaks, and cellular apoptosis (Chio et al., 2016), and in an *in vivo* model of transient whole-brain ischemia in rats, an increase in NOR1 mRNA expression levels in the hippocampus and piriform cortex were also observed (Kim et al., 2006). In a more recent study by Caba et al. (2021), researchers found that stimulation of the N-methyl-D-aspartic acid (NMDA) receptor by extracellular glutamate, overaccumulation of which is frequently observed in the pathogenesis of cerebral ischemia, triggered a rapid increase in NOR1 mRNA expression levels in hippocampal slice cultures. Further analysis showed that elevated NOR1 can relay neuronal survival signals by transcriptionally increasing the expression of cellular inhibitor of apoptosis protein 2 (cIAP2). Silencing of the NOR1 gene can suppress the oxygen-glucose deprivation (OGD)-induced increase in cIAP2 expression while enhancing hypoxia-induced neuronal damage (Chio et al., 2016). These results indicate that increased NOR1 expression or function may help to reduce the neuronal damage caused by cerebral ischemia. However, it is noteworthy that in some other studies, NOR1 was induced in cultured neurons of cerebral cortex only by depolarization but not by OGD. This suggests that hypoxic stimulation may not be sufficient to induce neuroprotective NOR1 and that depolarization signaling may also be important for the induction of NOR1 under conditions of cerebral ischemia. Future studies should clarify how ischemia, hypoxia, and depolarization affect NOR1 expression in the brain. Because an ischemic insult can induce brain-derived neurotrophic factor (BDNF) expression in the hippocampus, where NOR1 expression is also observed (Lindvall et al., 1992), it is reasonable to speculate that the increase in NOR1 expression induced by ischemia, hypoxia, or depolarization may be mediated by neurotrophic factors, which should be clarified by future studies.

**FIGURE 2:**
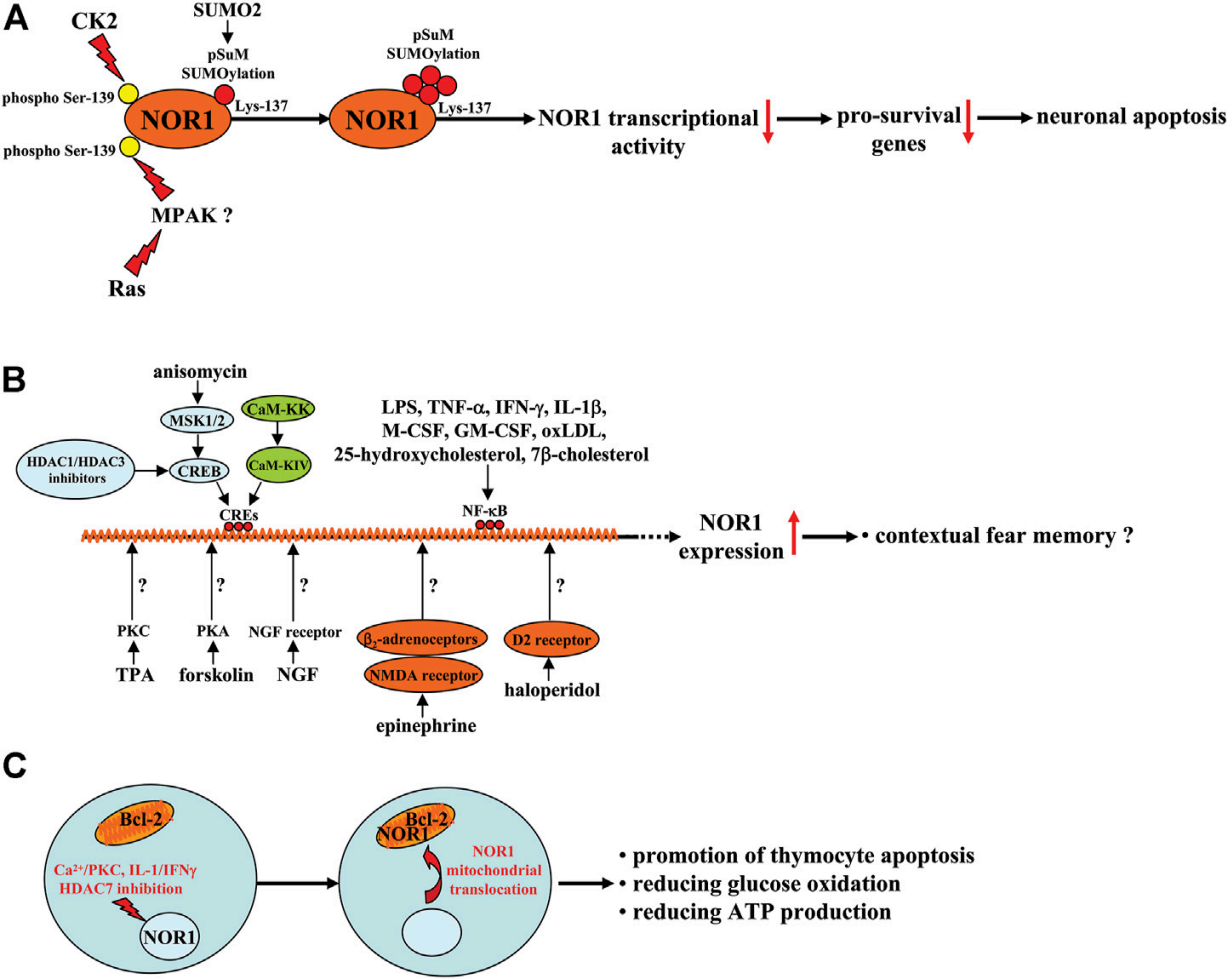
A schematic summary of the factors regulating NOR1 expression, function, and subcellular translocation in different cell types. **(A)** CK2 or Ras-MAPK signaling reduces NOR1 transcriptional activity by increasing SUMO2-mediated pSuM-SUMOlyation of NOR1 in a manner that depends on NOR1 phosphorylation at the Ser-139 site and leads to neuronal apoptosis. **(B)** Anisomycin-triggered MSK1/2-CREB signaling, CaM-KK-CaM-KIV signaling, and HDAC1 or HDAC3 inhibitors may bind to CREs in the NOR1 promoter and promote NOR1 transcription with unclear functions or a fear memory-promoting effect. Forskolin, TPA, or NGF promote NOR1 transcription by activating PKA, PKC, and the NGF receptors, respectively, although the exact molecular mechanisms are not yet clear. Epinephrine and haloperidol have been shown to increase NOR1 gene transcription probably by mobilizing β_2_-adrenoceptor-NMDA receptor signaling and D2 receptors, respectively. Factors such as LPS, TNF-α, IFN-γ, IL-1β, M-CSF, GM-CSF, oxLDL, 25-hydroxycholesterol, and 7β-cholesterol likely increase NOR1 expression through activation of NF-κB. **(C)** PKC activation, in combination with Ca^2+^, can induce mitochondrial translocation of NOR1 and exposure of the BH3 domain in Bcl-2, leading to thymocyte apoptosis. In INS cells, the pro-inflammatory cytokines IL-1/IFNγ can induce mitochondrial translocation of NOR1 and possible exposure of the BH3 domain in Bcl-2, leading to a decrease in glucose oxidation and ATP production. In thymocytes, when stimulated by signals from the TCR complex, HDAC7 could be phosphorylated and exported from the nucleus, promoting thymocyte apoptosis.

The above studies suggest that exploring drugs or factors that can regulate NOR1 expression or function may help to develop new strategies to alleviate damage caused by cerebral ischemia (the beneficial effects of NOR1 in the nervous system are shown in Figure 1). Indeed, this hypothesis has been explored in previously published studies. For example, casein kinase 2, a kinase involved in a variety of cellular processes, including cell survival, proliferation, apoptosis, and metabolism, has been shown to increase pSuM-SUMOylation (Lys-137) of NOR1 by increasing the phosphorylation level of NOR1 at its Ser-139 site, thereby decreasing NOR1 transcriptional activity and promoting cellular apoptosis in neurons (Gagnon et al., 2021; Figure 2A). Another kinase, Ras, can also decrease NOR1 transcriptional activity by increasing pSuM-SUMOylation (Lys-137) of NOR1 in a manner that is dependent on NOR1 phosphorylation at its Ser-139 site or MAPK (Gagnon et al., 2021; Figure 2A). Mitogen- and stress-activated protein kinase (MSK1) and MSK2 are kinases activated by either the extracellular signal-regulated kinase 1/2 (ERK1/2) or p38 mitogen-activated protein kinase (MAPK) pathway. They have been shown to mediate anisomycin-induced expression of NOR1 mRNA in primary mouse embryonic fibroblast cells by activating CREB and recruiting the co-activator CREB-binding protein (CBP) to cAMP-response element (CREs) (Darragh et al., 2005; Hawk and Abel, 2011) (Figure 2B). A Ca^2+^/calmodulin-dependent protein kinase IV (CaM-KIV) cascade response element located between 3,162 bp and 342 bp in the 1.7 kb NOR1 promoter and containing triple CREs was identified in previous studies, suggesting that NOR1 is a target gene of the CaM-KIV cascade (Inuzuka et al., 2002) (Figure 2B). Functional studies have shown that activation of the CaM-KK/CaM-KIV signaling cascade can increase the expression of NOR1 mRNA in SH-SY5Y cells (Inuzuka et al., 2002). Protein kinase A (PKA) activator forskolin, protein kinase C (PKC) activator 12-O-tetra-decanoyl-phorbol-13-acetate (TPA), or tyrosine kinase receptor B activator nerve growth factor (NGF) have been shown to induce NOR1 activation in various cell types, including PC12, C6, NIH3T3, L, GH, AtT-20, and Y-l cells (Bandoh et al., 1995) (Table 1; Figure 2B). Because inhibition of histone deacetylase 1 (HDAC1) and HDAC3 can increase NOR1 expression by recruiting phosphorylated CREB to the NOR1 promoter (Zhao et al., 2014; Figure 2B) and HDAC inhibition is widely known to have neuroprotective effects (Shukla and Tekwani, 2020), investigators should also examine whether CREB-triggered expression of NOR1 mediates the neuroprotective effects of HDAC inhibition.

**TABLE 1:** A summary of drugs or small molecules that can regulate NOR1 expression in various types of cells and tissues.

Drugs, small molecules	Cells/tissues, species	Effect on NOR1	Pharmacological effect	References
antipsychotic drugs: risperidone, chlorpromazine, fluphenazine, haloperidol, olanzapine, raclopride, risperidone, clozapine, quetiapine	striatum, nucleus accumbens, medial prefrontal cortex, ventral tegmental area, hippocampus; mouse	Up-regulation	Uncertain	Eells et al. (2012), Maheux et al. (2005)
epinephrine	hippocampus; mouse	Up-regulation	Mediating contextual fear memory	Oliveira et al. (2018), Martinho et al. (2020)
anisomycin	embryonic fibroblast cells; mouse	Up-regulation	Uncertain	Hawk and Abel (2011), Darragh et al. (2005)
TPA, LPS, TNF-α, IFN-γ, IL-1β, M-CSF, GM-CSF, oxLDL, 25-hydroxycholesterol, 7β-cholesterol	THP-1 cells, human monocyte-derived mouse macrophages, peritoneal macrophages, RAW264.7 cells, TPA-differentiated THP-1 cells; human, mouse	Up-regulation	Uncertain	McMorrow and Murphy (2011), Pei et al. (2005)
IL-1β	chondrocytes, rat	Up-regulation	Promotion of IκB-α degradation and NF-κB phosphorylation and nuclear translocation	Ma et al. (2020)
IL-4	peripheral blood mononuclear cells; human	Up-regulation	Promotion of the production of anti-inflammatory mediators	De Paoli et al. (2015)
Forskolin	PC12 cells, C6 glial cells, NIH3T3 cells, L cells, and GH, AtT-20, or Y-l cells	Up-regulation	Uncertain	Bandoh et al. (1995)
TPA				
NGF				
Scriptaid	aortic smooth muscle cells; rat	Up-regulation	Uncertain	Zhao et al. (2014)
PDGF	vascular smooth muscle cells; human, swine	Up-regulation	Promotion of vascular smooth muscle cell proliferation	Martínez-González et al. (2003); Bi et al. (2013)
Thrombin	vascular endothelial cells; human	Up-regulation	Promotion of endothelial growth	Martorell et al. (2007)
Ang II	vascular smooth muscle cells; rat, human	Up-regulation	Promotion of Giver-mediated oxidative stress and inflammation	Das et al. (2018); Cañes et al. (2020), Cañes et al. (2021)
	aortas; mouse, rat		Promotion of proinflammatory cytokine, chemokine, and reactive oxygen species production	
	arteries; hypertensive patients		Disrupting elastin integrity in vessels	
	vascular smooth muscle cells, mouse		Increasing AAA severity	
	cardiac tissues, mouse		Promotion of cardiac hypertrophy and fibrosis	
isoprenaline	cardiac tissues; mouse; liver, mouse	Up-regulation	Promotion of cardiac hypertrophy	Myers et al. (2009), Feng et al. (2015), Myers et al. (2009)
			Regulate lipogenesis and glucose homeostasis	
neosynephrine	pluripotent stem cell-derived cardiomyocytes (iPS-CM), mouse	Up-regulation	Mediating the neosynephrine-induced hypertrophy of iPS-CM	Philipp et al. (2021)
exendin-4	vascular smooth muscle cells; mouse	Down-regulation	Suppression of vascular smooth muscle cell proliferation	Takahashi et al. (2019)
miR-107	pulmonary artery smooth muscle cells; mouse, human pulmonary artery walls in acute pulmonary embolism; mouse, human	Down-regulation	Suppression of pulmonary artery muscle cell proliferation or migration	Chen et al. (2019), Chen L. et al. (2020), Li et al. (2022)
miR-106b-5p				
miR-34a-3p				
IL-1β	Islets	Up-regulation	Promotion of DNA breaking, cytochrome C release, β cell apoptosis	Close et al. (2013)
TNF-α				
LINC00467	hepatocellular carcinoma cells	Down-regulation	Promotion of tumor cell growth	Wang et al. (2020)
Z-ligustilide	AML cells	Up-regulation	AML inhibition	Wang C. et al. (2021), Zhou et al. (2013), Boudreaux et al. (2019)
SNDX-275 dihydroergotamine				
PGE1				
PGA2	NIH3T3 cells spleen cells	Up-regulation	Uncertain	Kagaya et al. (2005)
8-Br-cAMP glucagon	primary cultured hepatocytes or liver, mouse	Up-regulation	Involvement in regulation of lipogenesis and glucose homeostasis	Pei et al. (2006)
dietary restriction	liver, rat	Up-regulation	Involvement in regulation of lipogenesis and glucose homeostasis	Oita et al. (2009)

### Detrimental effect of NOR1 in the nervous system

NOR1 is not always considered neuroprotective, and under certain conditions NOR1 has been considered detrimental to the nervous system. For example, selective inhibition of NOR1 expression by antisense oligonucleotide was shown to promote cell migration, extension of processes, and formation of cellular aggregates in primary cultured forebrain cells (Ohkura et al., 1994), suggesting that NOR1 may have the ability to suppress neurite outgrowth. In addition, silencing the NOR1 gene in the hypothalamus was found to suppress food intake and body weight in mice (Nonogaki et al., 2009), suggesting that the existence of NOR1 in the hypothalamus might promote food intake and weight gain. Schaffer et al. (2010) had reported that forced expression of NOR1 in the amygadala can induce depression-like behaviors in rats, while the NOR1 gene deficiency elicits a reverse effect, suggesting that pathologically elevated NOR1 may mediate the pathogenesis of depression. This hypothesis is supported by another finding: restraint stress, which has been shown to trigger depression-like behaviors, leads to a significant increase in NOR1 mRNA expression levels in the adrenal pituitary and hypothalamus (Helbling et al., 2014) (Figure 1).

Epinephrine, a substance belonging to the catecholamines, has been shown to enhance contextual fear memories by acting on peripheral β_2_-adrenoceptors (Cahill and Alkire, 2003; Dornelles et al., 2007). Passively induced NOR1 expression could mediate the enhancing effect of epinephrine on contextual fear memories because 1) epinephrine can induce a significant increase in NOR1 mRNA expression in the hippocampus in mice likely through activation of β_2_-adrenoceptors and/or N-methyl-D-aspartic acid (NMDA) receptors (Figure 2B), which was reduced in epinephrine-deficient mice after fear conditioning (Oliveira et al., 2018), and 2) in a study investigating the necessary role of epinephrine in the persistence of traumatic memories in post-traumatic stress disorder, a concomitant increase in NOR1 mRNA expression was found in the hippocampus of mice (Martinho et al., 2020) (Table 1; Figures 1, 2B). These results are highly consistent with previous findings that NOR1 in the brain correlates with fear learning and memory. For example, researchers have found that contextual fear conditioning can induce a significant increase in NOR1 mRNA expression levels within 1 h in the hippocampus (Hawk et al., 2012), and this increase can be inhibited by disrupting CREB interaction with CBP (Bridi et al., 2017). However, to date, it is unclear how this increase is involved in the process of contextual fear memory. We found that the BDNF, Fos related/like antigen 2 (FOSL2), and p21-activated kinase 6 (PAK6) genes have potential NR4A binding sites in the proximal promoter region, and the BDNF gene may be a direct target of NR4A2 (Volpicelli et al., 2007). Therefore, NOR1 in the hippocampus likely mediates the process of learning and memory by regulating the expression of BDNF, FOSL2, and PAK6. This hypothesis is supported to some extent by some evidence: Inhibition of NR4A1 using the dominant-negative form of NR4A1 can downregulate mRNA expression of BDNF, FOSL2, and PAK6 in the brain (Bridi et al., 2017). Based on the review by Hawk and Abel (2011), which discussed the role of the NR4A transcription factor in learning and memory, researchers should identify the precise downstream target that mediates the involvement of NOR1 in learning and memory. It should also be noted that during fear memory formation, gene expression of the other two NR4A family proteins, NR4A1 and NR4A2, is also remarkably increased in the hippocampus (Hawk et al., 2012), suggesting that NOR1 is not the only protein mediating the process of contextual fear memory. The exact contributions of each NR4A protein to learning and memory need to be elucidated in future studies. The deleterious effects of NOR1 in the central nervous system are shown in Figure 1.

### Uncertainties of NOR1 in the nervous system

Although NOR1 is expressed in different regions of the brain, NOR1 mRNA expression has been found to be deficient in dopaminergic neurons in the substantia nigra and ventral tegmental area of the midbrain (Zetterström et al., 1996). This suggests that the changes in NOR1 expression in dopaminergic neurons may have potential significance in regulating signals related to dopaminergic neurotransmission. Haloperidol, an antagonist of the D2 receptor used to treat schizophrenia, can rapidly upregulate NOR1 mRNA expression levels in the ventral tegmental area of mice, followed by a significant increase in tyrosine hydroxylase and dopamine transporter expression levels (Table 1; Figure 2B) (Eells et al., 2012). Antipsychotics such as risperidone, chlorpromazine, fluphenazine, haloperidol, olanzapine, raclopride, risperidone, clozapine, and/or quetiapine have been shown to rapidly increase NOR1 mRNA expression levels in the striatum, nucleus accumbens shell and core, or medial prefrontal cortex of mice (Table 1) (Maheux et al., 2005). In addition, the antipsychotic drugs haloperidol, raclopride, clozapine, risperidone, and olanzapine can increase NOR1 mRNA expression in the hippocampus of mice (Table 1) (Maheux et al., 2005). These results indicate that NOR1 can be dynamically regulated in dopamine neurons and may be a potential target for intracellular signaling events related to dopaminergic neurotransmission. Further experiments should be performed to confirm whether NOR1 induced by antipsychotic drugs is beneficial or harmful (Figure 1).

Another study reported that NOR1 expression in the cerebral cortex, piriform cortex, amygdala, and hippocampus of mice can be induced by stimuli associated with increased neuronal activity, such as 1-h electrical stimulation and brief exposure to a novel environment (Sun et al., 2007). *In vitro* studies have shown that KCl-induced depolarization can trigger a significant increase in NOR1 mRNA expression in cultured hippocampal and cortical neurons (Sun et al., 2007). These results indicate that NOR1 may be a useful marker reflecting neuronal activity. However, whether these neuronal activity-dependent increases in NOR1 expression are beneficial or detrimental remains unclear (Figure 1).

Researchers also observed NOR1-positive signals in neurons and Schwann cells in the spinal cord of patients suffering from Lewy body disease but not tauopathies, Tar DNA binding protein 43 kDa (TDP-43) proteinopathies, and polyglutamine disorders, suggesting that pathologically elevated NOR1 in α-synucleinopathy may be involved in the pathogenesis of neurodegenerative diseases (Kon et al., 2015). But to date, the relationship between NOR1 and α-synucleinopathy is unclear. That is, we still do not know whether the accumulation of NOR1 in α-synucleinopathy is beneficial or detrimental (Figure 1). Further exploration of this question may help to develop new targets for the treatment of Parkinson’s disease and dementia with Lewy bodies.

### The role of NOR1 in pathophysiological process in immune and inflammatory cells

Apoptosis associated with negative thymocyte selection is a central event in T cell development. NOR1 has been shown to be associated with the development and regulation of thymocyte function. The expression of NOR1 has been shown to be induced in thymocytes at a high level, which subsequently triggers thymocyte apoptosis (Cheng et al., 1997; Gao et al., 2022), suggesting that NOR1 may play a key role in the clonal deficiency of autoreactive thymocytes (Sohn et al., 2007). Induction of the expression of CD25, a pro-apoptotic protein, may be a possible mechanism for the pro-apoptotic effect of NOR1 in thymocytes (Cheng et al., 1997; Gao et al., 2022). Further analysis showed that stimulation of CD4^+^CD8^+^ thymocytes may lead to translocation of NOR1 from the nucleus to the mitochondria. Mitochondrial NOR1 then binds to B-cell lymphoma-2 (Bcl-2) and induces a conformational change of the BH3 domain in Bcl-2, leading to thymocyte apoptosis (Thompson and Winoto, 2008; Odagiu et al., 2021) (Figure 2C), suggesting that the pro-apoptotic conversion of Bcl-2 may be a possible mechanism for the promotion of thymocyte apoptosis by NOR1. Moreover, PKC activation in combination with Ca^2+^ has been shown to trigger mitochondrial translocation of NOR1 and release of the BH3 domain in Bcl-2, thereby promoting thymocyte apoptosis (Figure 2C) (Thompson et al., 2010). Researchers have also investigated the mechanisms underlying the regulation of NOR1 expression by various factors during thymocyte preselection. In preselected thymocytes, NOR1 expression was found to be suppressed by HDAC7 (Figure 2C) (Oh et al., 2016), suggesting that the integrity of HDAC7 activity may be required for the suppression of thymocyte apoptosis and that inhibition of HDAC7 may promote thymocyte apoptosis. Specifically, when stimulated by signals from the T cell receptor (TCR) complex, HDAC7 could be phosphorylated and exported from the nucleus (Figure 2C) (Dequiedt et al., 2005; Parra et al., 2005), disrupting its repressive effect on the NOR1 gene and promoting thymocyte apoptosis. This hypothesis may be supported by another finding: Transgenic mice carrying an HDAC7 mutant, which is presumably unable to be phosphorylated downstream of TCR signaling and exported from the nucleus, show impaired induction of NOR1 as well as impaired negative selection and lethal autoimmunity (Kasler et al., 2012).

Regulatory T cells (Tregs) are a specific type of T cell. They can inhibit the activity and functions of effector T cells by targeting autogenous and non-autogenous antigens, which are critical for maintaining immune homeostasis (Grover et al., 2021). NOR1 may play a key role in Treg cell development, as researchers have found that a combined deletion of NR4A1 and NOR1 is associated with a significantly greater amount of peripheral Treg cells, while a triple deletion of NR4A1, NR4A2, and NOR1 (NR4A3) has a negative effect on Treg cell development (Sekiya et al., 2013). Further analysis showed that NOR1 regulates the development of Tregs by activating the transcription factor Forkhead Box P3 (Foxp3) (Bandukwala and Rao, 2013; Sekiya et al., 2013), suggesting that NOR1 is required in part for the regeneration of Foxp3 Treg cells. Dendritic cells are antigen-presenting cells in the immune system. The mature dendritic cells can migrate to the draining lymph nodes and activate the antigen-specific T cells (Yamazaki and Morita, 2013). NOR1 appears to be essential for the homeostatic mitochondrial function of CD103^+^ dendritic cells and the migration of CD103^+^ dendritic cells to lymph nodes, as it has been shown that NOR1 deficiency induces lower basal respiration in NOR1-deficient CD103^+^ dendritic cells and loss of CD103^+^ migrating dendritic cells in the lymph node in NOR1-deficient mice in a manner that is dependent on the existence of the membrane chemokine CCR7 (Park et al., 2016). Overall, NOR1 can simultaneously promote thymocyte apoptosis and Treg cell regeneration. However, the exact reason for the differential effect of NOR1 in different immune cells remains to be determined.

**FIGURE 3:**
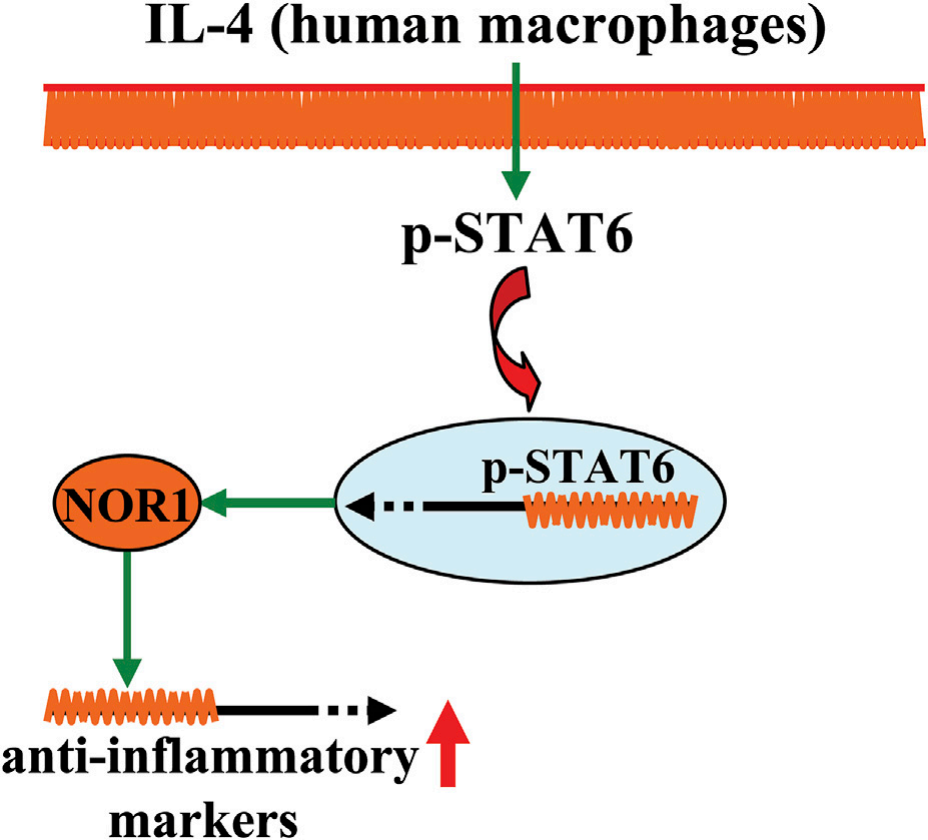
A schematic showing the regulatory effect of NOR1 in regulating inflammation in macrophages. In human macrophages, NOR1 appears to mediate the production of anti-inflammatory factors triggered by IL-4, which may contribute to the resolution of inflammation.

Inflammation is an adaptive biological response to infection, injury, and autoimmune processes. Although acute inflammation can promote recovery of tissue structure and function, chronic inflammation is closely associated with the development of chronic disease. There is increasing evidence that NOR1 may be involved in the regulation of inflammation (Table 1) (Pei et al., 2005; McMorrow and Murphy, 2011). For example, molecules that stimulate pro-inflammatory responses, such as TPA, lipopolysaccharide (LPS), tumor necrosis factor-α (TNF-α), interferon-γ (IFN-γ), interleukin-1β (IL-1β), macrophage colony-stimulating factor (M-CSF), and granulocyte-macrophage colony-stimulating factor (GM-CSF), have been shown to increase NOR1 mRNA expression, possibly by inducing IκB-α degradation and thereby inhibiting nuclear factor-κB (NF-κB) signaling in cultured THP-1 cells, primary human monocyte-derived macrophages, isolated mouse peritoneal macrophages, and RAW264.7 cells (Pei et al., 2005; McMorrow and Murphy, 2011). NOR1 mRNA can also be induced by various types of oxysterols associated with the development and progression of inflammation-related disorders, such as oxidized low-density lipoprotein (oxLDL), 25-hydroxycholesterol, and 7β-cholesterol, in THP-1 cells, TPA-differentiated THP-1 cells, and primary human monocyte-derived macrophages (McMorrow and Murphy, 2011; Pei et al., 2005; Figure 2B). These results indicate that NOR1 may be a key protein mediating the processes of pro-inflammatory responses. However, it is worth mentioning that there are also other voices for the pro-inflammatory effect of NOR1 especially in macrophages (Rodríguez-Calvo et al., 2017). A study by De Paoli et al. (2015) had reported that the existence of NOR1 could favor the production of anti-inflammatory markers in human macrophages. The human NOR1 promoter has several responsive elements that can respond to the IL-4 signal: IL-4 first activates the signal transducer and activator of transcription 6 (STAT6), and then the activated STAT6 directly binds to the NOR1 promoter in IL-4-stimulated human macrophages, inducing the expression of NOR1 and promoting the production of anti-inflammatory markers, such as interleu-kin-1 receptor antagonist (IL-1Ra), CD200 receptor, and IL-10 (De Paoli et al., 2015) (Figure 3) (Table 1). Silencing of the NOR1 gene significantly suppresses IL-4-induced production of anti-inflammatory markers (De Paoli et al., 2015). *In vivo* studies had reported that NOR1 is strongly expressed in the region containing CD68^+^/MR^+^ M2 macrophages in human atherosclerotic plaques (De Paoli et al., 2015). Another study by Bonta et al. showed that NOR1 mRNA can be checked in macrophages with atherosclerotic lesions and that overexpression of NOR1 reduces the expression and production of IL-1β, IL-6, IL-8, macrophage inflammatory protein-1, and monocyte chemoattractant protein-1 (MCP-1) in cultured THP-1 cells, whereas silencing of the NOR1 gene has an opposite effect (Bonta et al., 2006). These results indicate that NOR1 may exert an anti-inflammatory function by shifting macrophages toward an anti-inflammatory phenotype. At this stage, it is still unclear why NOR1 has simultaneous proinflammatory and anti-inflammatory functions. Further exploration of the role of NOR1 in regulating inflammation may pave the way for the development of novel strategies to regulate inflammatory cells.

### The role of NOR1 in the cardiovascular system

Cardiovascular disease is prevalent in modern society and can impose severe social and economic burden. The search for new targets that can be used to develop drugs for cardiovascular disease is of great clinical importance. Researchers have found that the expression levels of NOR1 are remarkably increased in proliferated and remodelled vessels (Rodríguez-Calvo et al., 2013; Martínez-González et al., 2021), suggesting that NOR1 may be a risk factor for promoting cardiovascular diseases that are closely associated with inflammation (Hafiane and Daskalopoulou, 2022). An enhanced inflammatory response is an important pathological event that mediates the progression of cardiovascular disease. In the above sections, we mentioned that NOR1 has anti-inflammatory effects in human macrophages (De Paoli et al., 2015); thus, NOR1 might also contribute to the alleviation of cardiovascular disease. Here, we systematically summarize and discuss the role of NOR1 in different types of cardiovascular diseases and the factors that may regulate NOR1 expression or function in the cardiovascular system to provide a comprehensive understanding of the role of NOR1 in the cardiovascular system.

### The role of NOR1 in atherosclerosis

**FIGURE 4:**
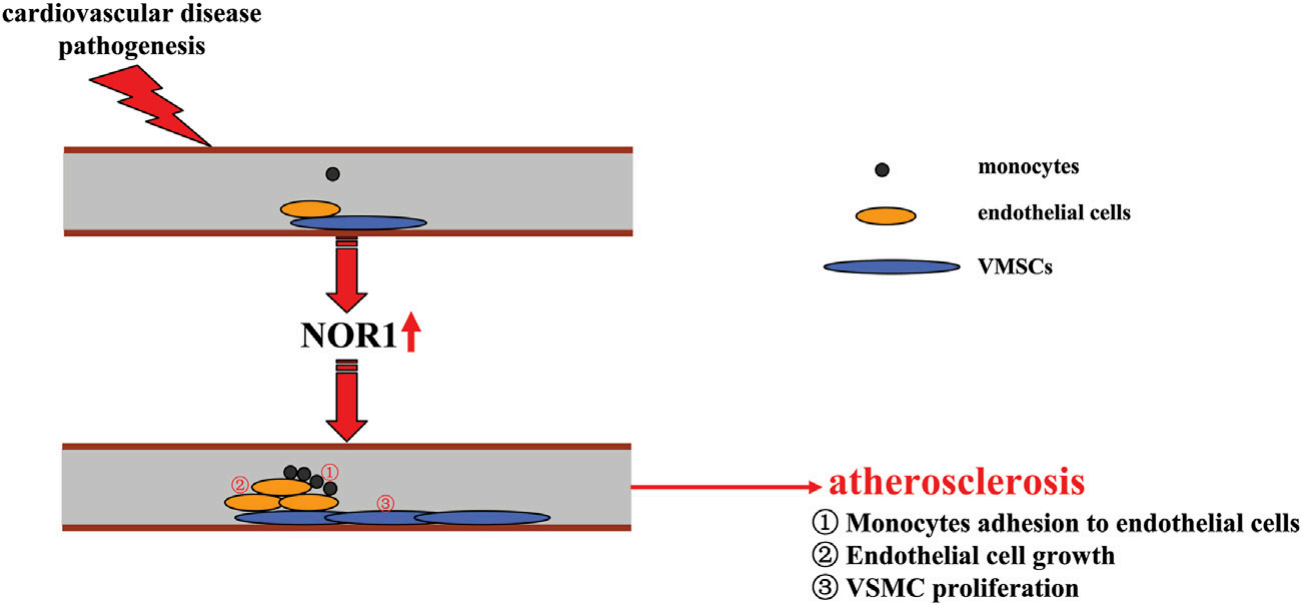
A schematic summary of how NOR1 might be involved in the development of cardiovascular diseases such as atherosclerosis. Pathologically elevated NOR1 could promote the development of atherosclerosis by inducing adhesion of monocytes to endothelial cells, increasing endothelial cell growth, and promoting proliferation of VSMCs.

Atherosclerosis is a common cardiovascular disease in modern society. Macrophages play a critical role in the development of atherosclerosis. Conversion of overactivated macrophages to an anti-inflammatory phenotype may be beneficial for the treatment of atherosclerosis. NOR1 may be involved in regulating monocyte adhesion to endothelial cells by regulating the expression of vascular cell adhesion molecule-1 (VCAM-1) and intercellular cell adhesion molecule-1 (ICAM-1). NOR1 deficiency reduces hypercholesterolemia-induced atherosclerosis formation in apoE (−/−) mice by decreasing the macrophage content of the lesion (Zhao et al., 2010) (Figure 4). In addition to adhesion between monocytes and endothelial cells, proliferation of endothelial cells is another important pathological event involved in the angiogenic processes associated with cardiovascular diseases such as atherosclerosis. The regulation of endothelial cell growth may be of importance for the development of strategies to treat cardiovascular disease. In a previous study, thrombin, a substance that promotes blood coagulation, was found to trigger early and transient upregulation of NOR1 by acting on its receptor protease-activated receptors-1 (PAR-1) and subsequent Ca^2+^-PKC-MAPK signaling, which may lead to promotion of vascular endothelial cell growth (Martorell et al., 2007) (Table 1). CREB signaling also appears to be critical to this process, as inhibition of PAR-1 can abrogate CREB-mediated upregulation of NOR1 expression in vascular endothelial cells, and the NOR1 promoter has two CRE sites in the -79 and -53 bp that can respond to CREB (Martorell et al., 2007; Martorell et al., 2008). These effects of NOR1 show that NOR1 could be a molecule that can mediate vascular endothelial cell growth (Figure 4) and strategies that block NOR1 expression or function may be beneficial for the treatment of cardiovascular diseases associated with pathological changes in vascular endothelial cells.

Vascular smooth muscle cell proliferation is also a key event in the pathogenesis of atherosclerosis. Under normal conditions, VSMCs are in a quiescent state and do not migrate, whereas under conditions stimulated by growth factors and cytokines, these cells proliferate and migrate rapidly, which subsequently contributes to the pathogenesis of atherosclerosis. Recent studies had reported that platelet-derived growth factor (PDGF), which plays a critical role in the pathogenesis of atherosclerosis, can induce the expression of NOR1 in a manner dependent on ERK1/2-MAPK and CREB signaling, and that the increased NOR1 can mediate the subsequent proliferation of VSMCs (Martínez-González et al., 2003; Bi et al., 2013) (Figure 4; Table 1). Further analysis revealed that NADPH oxidase 1 (NOX1) is a downstream target of NOR1 and may mediate the regulatory effects of NOR1 in vascular remodeling by regulating the oxidation-reduction response in human VSMCs: 1) overexpression of NOR1 can increase reactive oxygen species (ROS) production and NOX1 expression in human VSMCs, and 2) silencing of the NOX1 gene can counteract the increased ROS production and cell migration induced by NOR1 in human VSMCs (Alonso et al., 2018). The regulation of NOX1 by NOR1 in VSMCs may be mediated by Giver, a protein whose knockdown can attenuate the expression of genes involved in NOX1 expression and proinflammatory cytokine production. Angiotensin II (Ang II) has been shown to increase the expression of Giver by recruiting NOR1 to the Giver promoter in VSMCs, and Giver and NOR1 were observed to be induced in Ang II-treated human VSMCs and in arteries from hypertensive patients (Das et al., 2018) (Table 1). These results suggest that NOR1 is a molecule that can promote vascular smooth muscle cell proliferation. Strategies that block NOR1 expression or function may contribute to the treatment of atherosclerosis associated with VSMC proliferation.

Notably, however, NOR1 has also been shown to mediate anti-atherosclerotic effects in several ways. For example, overexpression of NOR1 has been shown to suppress the LPS-induced increase in the expression of IL-1β, IL-6, MCP-1, and CC chemokine ligand 20 (CCL20) by increasing the transcriptional activity of NF-κB-sensitive promoters in human VSMCs (Calvayrac et al., 2015). NOR1 may also mediate the pro-angiogenic response to vasoconstrictor peptides (Wang C. et al., 2021). It has been observed that NOR1 expression is induced in endothelial cells exposed to hypoxia in a hypoxia-inducible factor 1 (HIF-1)-dependent manner, which likely mediates the adaptive survival response of endothelial cells to hypoxia (Martorell et al., 2009). NOR1 expression can be induced by an anti-inflammatory mediator IL-4, and silencing of the NOR1 gene can reduce the expression of anti-inflammatory markers (De Paoli et al., 2015). In human atherosclerotic plaques, NOR1 is observed in alternative macrophages (De Paoli et al., 2015). In general, these findings indicate that NOR1 can attenuate the pathogenesis of atherosclerosis through mechanisms such as alleviating inflammation and protecting endothelial cells. However, the exact reasons for the conflicting positive and negative effects of NOR1 on the pathogenesis of atherosclerosis should be carefully investigated in future studies.

### The role of NOR1 in abdominal aortic aneurysm

Abdominal aortic aneurysm (AAA) is a cardiovascular disease associated with pathological events such as VSMC apoptosis (Nordon et al., 2011; Galán et al., 2016). To date, there are no effective therapies for this life-threatening disease. Therefore, there is an urgent need to search for new targets for the development of novel drugs to treat AAA. In studies using VSMCs in AAA, researchers have observed a significant increase in the expression levels of NOR1 protein in human AAA samples, which is mainly localized in VSMCs (Alonso et al., 2016). *In vitro* studies have shown that overexpression of NOR1 can limit VSMC apoptosis (Alonso et al., 2016). Because VSMC apoptosis is a key factor in the progression of AAA (Nordon et al., 2011; Galán et al., 2016), this finding indicates that the increased NOR1 protein observed in samples from AAA may counteract the deleterious changes induced by AAA-triggering stimuli. However, some other studies yielded different, contradictory results. For example, studies by Cañes et al. (2021) showed that overexpression of human NOR1 in the vasculature of mice, particularly in VSMCs, enhances Ang II-induced production of proinflammatory cytokines, chemokines, and reactive oxygen species, Ang II-induced increase in matrix metalloproteinase activity, and Ang II-induced disruption of elastin integrity in vascular tissue, which subsequently increases the occurrence and severity of AAA (Table 1). Because this finding provides for the first time direct results showing a clear association between VSMC NOR1 and the pathogenesis of AAA, it is reasonable to hypothesize that abnormally increased NOR1 may contribute to the pathological progression of AAA, at least in animal models of AAA. The search for drugs or other strategies that block the function or expression of NOR1 may be useful for the treatment of AAA.

It is well known that the pathogenesis of AAA is associated with inflammation (Koch et al., 1990; Saraff et al., 2003). Overexpression of NOR1, which increases inflammatory responses in VSMCs, provides direct results showing the association between inflammation and AAA (Cañes et al., 2021). However, it is worth noting that this evidence was obtained only in VSMCs, which generally do not directly demonstrate the association between inflammation and AAA. In fact, some other studies show that inflammation in different cell types contributes to the pathogenesis of AAA in different ways. For example, studies by Qing et al. (2017) showed that although NOR1 deficiency in hematopoietic stem cells can block the LPS-induced pro-inflammatory response in macrophages, this alteration had no effect on the development of AAA formation in bone marrow-derived stem cell transplanted mice with low-density lipoprotein receptor deficiency, suggesting that macrophage-derived inflammation does not contribute to the development of AAA. The cell-specific contribution of the different cell types involved in inflammation to the pathogenesis of AAA should be investigated in future studies.

### The role of NOR1 in cardiac hypertrophy

NOR1 was detected not only in VSMCs but also in heart samples from pigs, mice, and humans. Hypertensive cardiac hypertrophy is a common cause of heart failure, but to date, the precise molecular mechanisms underlying this pathogenesis remain largely unknown. Previous studies had reported that the mice with cardiac NOR1 overexpression had a significant increase in cell surface area in cardiomyocytes and expression of myosin heavy chain 7 (Cañes et al., 2020). Compared with cardiac fibroblasts in wide-type mice, cardiac fibroblasts in Tg NOR1 mice exhibit high levels of cardiac fibroblast markers and a strong capacity for collogen synthesis. NOR1 is also associated with Ang II-induced cardiac hypertrophy: Ang II can increase the expression of NOR1 in cardiac tissues; overexpression of NOR1 can exacerbate Ang II-induced cardiac hypertrophy and cardiac fibrosis (Cañes et al., 2020) (Table 1). Some other studies have reported that NOR1 is required for the induction of neosynephrine-induced cardiac hypertrophy (Philipp et al., 2021) (Table 1), and isoprenaline, another β-adrenergic agonist, has been found to induce NOR1 expression in mouse cardiac tissue, although no functional relationships have been established (Myers et al., 2009) (Table 1). NOR1 may also be involved in the process of isoprenaline-induced cardiac hypertrophy by regulating poly ADP-ribose polymerase-1 (PARP-1), and silencing of the NOR1 gene ameliorates neosynephrine-induced cardiac hypertrophy (Feng et al., 2015; Medzikovic et al., 2019) (Table 1). These results strongly suggest that NOR1 may be involved in the pathogenesis of hypertensive cardiac hypertrophy. However, it is particularly noteworthy that the pro-survival effect of NOR1 in cardiomyocytes, especially under the condition of doxorubicin (DOX) treatment, may also have beneficial effects (Berg et al., 2021). DOX is a widely used anticancer drug known to cause cardiotoxicity due to its promoting effect on ROS production. Researchers have found that overexpression of NOR1 can suppress DOX-induced death and apoptosis of cardiomyocytes (Berg et al., 2021). Thus, NOR1 may serve as a potential cardioprotective protein in response to DOX-induced cellular stress.

### Factors that may regulate NOR1 expression or function in the cardiovascular system

The above studies suggest that the regulation of NOR1 expression or function may contribute to the development of new strategies for the treatment of cardiovascular diseases. Indeed, this topic has been investigated in some previously published studies. For example, the glucagon-like peptide-1 (GLP-1) receptor agonist exendin-4 was shown to suppress vascular injury-induced neointima by suppressing NOR1 expression (Takahashi et al., 2019). *In vitro* studies showed that exendin-4 downregulates NOR1 expression in primary cultured VSMCs by inhibiting the phosphorylation of MAPK and CREB mediated by extracelluar signals (Takahashi et al., 2019). Pharmacological inhibition of HDAC by Scriptaid has been shown to increase NOR1 expression in rat aortic smooth muscle cells by recruiting phosphorylated CREB to the NOR1 promoter (Zhao et al., 2014) (Table 1). In addition, several microRNAs have been reported to regulate NOR1 expression in pulmonary artery smooth muscle cells (PASMCs). For example, miR-34a-3p, which is downregulated in rats and patients with acute pulmonary embolism (APE), was shown to suppress PDGF-induced expression of NOR1 in human PASMCs (Table 1) (Li et al., 2022). The miR-106b-5p, which was downregulated in PDGF-induced mouse PASMCs and APE mice, can suppress PDGF-induced proliferation and migration of mouse PASMCs by targeting the 3′ untranslated region of NOR1 mRNA and reducing the expression of NOR1 (Table 1) (Chen L. et al., 2020). Another microRNA miR-107 has been shown to suppress PDGF-induced proliferation and migration of human PASMCs by targeting the 3′ untranslated region of NOR1 mRNA and reducing the expression of NOR1 (Table 1) (Chen et al., 2019).

### The role of NOR1 in the digestive system

#### The role of NOR1 in the liver

Liver regeneration is an important process in maintaining homeostasis of liver function after partial hepatectomy. The expression of NOR1 has been shown to be significantly increased in the later initial and proliferation phases of liver regeneration, suggesting that NOR1 has the ability to promote the regeneration of liver cells, including hepatocytes and hepatic stellate cells (Vacca et al., 2013; Herring et al., 2019). Gene profile analysis revealed that NOR1 is involved in regulating the expression of genes related to cell cycle and proliferation in liver cells, such as cyclin D1, by directly interacting with the promoter regions of the cyclin D1 gene. In stellate liver cells, transforming growth factor-β1 (TGF-β1), a key molecule for cell activation and proliferation, has been shown to promote NOR1 expression, and suppression of NOR1 can inhibit TGF-β1-induced proliferation of stellate liver cells (Herring et al., 2019), suggesting that NOR1 may be critical for TGF-β1-mediated activation and proliferation of stellate liver cells. Since excessive proliferation of hepatic cells is closely associated with liver fibrosis, inhibition of NOR1 expression or function may be beneficial for the treatment of liver fibrosis.

In addition to regulating hepatic cell proliferation, NOR1 has also been shown to regulate the expression of genes related to lipogenesis and glucose homeostasis. For example, a single intraperitoneal injection of isoprenaline (20 mg/kg, between 1 and 4 h) in mice was found to rapidly induce the expression of NOR1 mRNA in the liver, along with an increase in the expression of a variety of genes, such as fatty acid synthase (Fasn), interleukin 15, fatty acid binding protein 4 (Fabp4), insulin receptor substrate 1 (Irs-1), insulin receptor, pyruvate dehydrogenase kinase (Pdk), leptin receptor (Lepr), glucose transporter 8 (Glut8), and signal transducers and activator of transcription (Stat3) gene (Myers et al., 2009). Researchers also found that dietary restriction up-regulated NOR1 gene expression in the livers of 28-month-old rats, along with a significant increase in peroxisome proliferator-activated receptor-α (PPAR-α) expression and a significant decrease in PPAR-β/δ expression (Oita et al., 2009). In addition, the stable cyclic adenosine monophosphate (cAMP) analog 8-Br-cAMP, known to activate the gluconeogenic program, was shown to induce a rapid and significant increase in NOR1 mRNA expression in primary cultured hepatocytes *via* activation of CREB (Pei et al., 2006). Under *in vivo* conditions, both fasting and glucagon administration induced a significant increase in NOR1 gene expression levels in mouse liver, accompanied by a concomitant increase in genes associated with the gluconeogenic pathway, such as glucose-6-phosphatase, catalytic (G6pc) and enolase 3 (Pei et al., 2006). Because the above genes are associated with lipogenesis and glucose homeostasis, it is reasonable to hypothesize that the induction of NOR1 in the liver, by whatever factor, may be related to the modulation of lipid and glucose metabolism.

The importance of the above findings should first be emphasized at the physiological level, because stimuli such as restricted dieting or fasting are well-known and widely studied factors that can modulate lipogenesis and glucose metabolism and are also considered potential strategies to prevent or treat metabolic disorders (Takeuchi et al., 2016; Schwarz et al., 2017). Further studies should be conducted to evaluate the precise contribution of NOR1 to the protective effects of fasting or dietary restriction. At the pathological level, the increase in NOR1 expression in the liver should also be reinforced, because researchers also observed a significant increase in NOR1 mRNA expression in the liver in db/db mice and in a mouse model of type II diabetes induced by streptozotocin treatment, and partial inhibition of NOR1 activity by Nur77-M1 (an activation domain mutant of Nur77) in db/db mice can cause a significant decrease in both fasting and random-fed blood glucose levels (Pei et al., 2006). In general, inhibition of NOR1 function or expression may contribute to the treatment of metabolic disorders, and NOR1 may be a potential target for drug development to treat metabolic disorders. However, because Nur77-M1 can also inhibit the activity of the other two NR4A family proteins in hepatocytes (Pei et al., 2006), further studies should be performed to determine the causal relationship between NOR1 in the liver and metabolic disorders related to the imbalance of lipogenesis and glucose homeostasis.

### The role of NOR1 in the pancreas

In the islet, pancreatic cell fate may be determined by the balance between endoplasmic reticulum stress and unfolded protein response. Thapsigargin and palmitate, two ER stress triggers, can increase the expression of NOR1 in a mouse pancreatic β-cell line, MIN6 cells, possibly contributing to the down-regulation of insulin secretion by down-regulating the expression of the insulin-positive-regulatory genes pancreatic and duodenal homeobox factor-1 (PDX-1) and neurogenic differentiation 1 (NeuroD1) (Gao et al., 2014). Pathologically elevated NOR1 in pancreatic β-cells may also have a pro-apoptotic effect: the proinflammatory cytokines IL-1β and TNF-α have been shown to stimulate NOR1 expression in cultured islets, along with DNA degradation, cytochrome C release, and β-cell apoptosis (Table 1) (Close et al., 2013). Another study had reported that NOR1 may be a hopeful target for developing strategies that can protect pancreatic β-cells in type II diabetes (Close et al., 2020). In this study, NOR1 expression was shown to be elevated in human islet cells and INS cells due to increased pro-inflammatory cytokines and glucose levels (Close et al., 2020). Moreover, overexpression of NOR1 has been reported to induce cellular apoptosis in INS and human islet cells, while silencing of the NOR1 gene blocks cytokine-triggered death of pancreatic β-cells. More importantly, the expression levels of NOR1 are significantly increased in islet cells from patients suffering from type II diabetes (Close et al., 2020). Mechanistic studies have shown that disruption of the mitochondrial network is the major pathway through which NOR1 affects islet cell and INS cell functions: under physiological conditions, NOR1 resides mainly in the cytoplasm, whereas upon treatment with pro-inflammatory cytokines, NOR1 can enter the mitochondria, where NOR1 reduces glucose oxidation and the production rate of adenosine triphosphate (ATP) (Figure 2C) (Close et al., 2020). These results indicate that NOR1 may be able to promote islet cell damage in metabolic dysfunction. A better understanding of the role of NOR1 in the pathophysiological process of type II diabetes may help to develop new strategies for the treatment of type II diabetes.

### The role of NOR1 in osteoarthritis and rheumatoid arthritis

**FIGURE 5:**
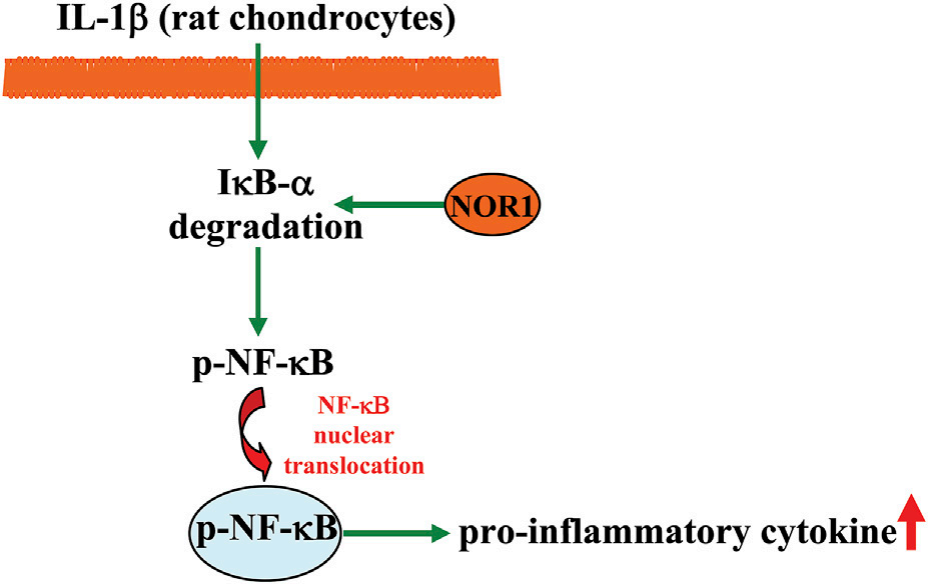
A schematic showing the regulatory effect of NOR1 in regulating inflammation in chondrocytes. The NOR promotes IL-1β-induced degradation of IκB-α and NF-κB nuclear translocation, thereby promoting inflammatory responses in rat chondrocytes.

Osteoarthritis is a common degenerative joint disease with severe cartilage loss (Cai et al., 2021). Since synovial inflammation is considered an important mechanism contributing to the progression of osteoarthritis, anti-inflammation is considered an important strategy for the treatment of osteoarthritis (Benito et al., 2005). In a previous *in vivo* study, researchers observed a significant increase in NOR1 expression in human osteoarthritis cartilage (Ma et al., 2020). In *in vitro* studies, overexpression of NOR1 was found to promote Earle’s balanced salt solution-induced chondrocyte apoptosis, which simultaneously correlated with the enhancement of NF-κB-dependent expression of pro-inflammatory mediators, including matrix metallopro-teinase-3 (MMP-3), MMP-9, cyclooxygenase-2 (COX-2), and inducible nitric oxide synthase (iNOS); in contrast, knockdown of the NOR1 gene suppressed EBSS-induced chondrocyte apoptosis (Table 1) (Ma et al., 2020). Moreover, overexpression of NOR1 can promote IL-1β-induced degradation of inhibitor kappa B-α (IκB-α) as well as NF-κB phosphorylation and nuclear translocation in rat chondrocytes, whereas silencing of the NOR1 gene has an opposite effect (Ma et al., 2020) (Figure 5). In *in vivo* studies, intra-articular injection of lentivirus to overexpress NOR1 was found to induce a pro-inflammatory and pro-apoptotic effect in a rat model of osteoarthritis, whereas down-regulating of NOR1 expression using the shRNA technique had an opposite effect (Ma et al., 2020). These results indicate that NOR1 likely mediates the pathogenesis of osteoarthritis in rats by enhancing a pro-inflammatory effect in a manner dependent on NF-κB signaling, and that suppression of NOR1 function or activity may represent a potential strategy for the treatment of osteoarthritis.

In another study by Andreas et al. (2009), researchers also observed a significant increase in NOR1 expression in human synovial fibroblasts in rheumatoid arthritis that was reversed by treatment with antirheumatic drugs, including azathioprine, chloroquine phosphate, gold sodium thiomalate, and methotrexate. This is the first report showing an association between pathologically increased NOR1 levels in human synovial fibroblasts in rheumatoid arthritis and antirheumatic treatment. Therefore, it is reasonable to hypothesize that NOR may be a promising target for the development of new antirheumatic drugs and that suppression of NOR1 function or activity may be a potential strategy for the treatment of rheumatoid arthritis. Futures studies should further clarify the role of NOR1 in the pathogenesis of rheumatoid arthritis.

### The role of NOR1 in tumor regulation

Cancer is both a medical and a public health problem. However, to date, most therapeutic drugs for various cancers have been consistently frustrating. The search for new targets for the development of new antitumor drugs is of great clinical importance. In most cancers, highly expressed NOR1 can exert tumor suppressive activities (Safe and Karki, 2021). For example, NOR1 can mediate the suppression of non-small cell lung cancer (NSCLC) cell growth induced by BRE-AS1 (a novel lncRNA BRE antisense RNA 1) (Zhang et al., 2018), and silencing of the NOR1 gene reduces the suppressive effect of radio-hyperthermia on A549 lung cancer cell proliferation (Son et al., 2019). In hepatocellular carcinoma (HCC), NOR1 expression shows a negative correlation with LINC00467, a long non-coding RNA that can promote tumor cell growth: LINC00467 was found to suppress the formation of post-transcriptional double-stranded RNA by interacting with and inducing the degradation of NOR1 mRNA, thereby promoting the abnormal growth of HCC cells (Table 1) (Wang et al., 2020). Restoration of NOR1 expression can block the pro-carcinogenic effect of LINC00467 (Wang et al., 2020). In acinar cell carcinoma, the specific re-composition phenotype [t (4; 9) (q13; q31)] may increase the expression levels of NOR1, thereby promoting the progression of salivary gland acinar cell carcinoma by up-regulating NOR1-regulated genes (Haller et al., 2019).

Mullican et al. had reported that there are abnormal phenotypes in Nur77/NOR1 gene double knockout mice (Nur77/NOR1 double −/−): The Nur77/NOR1 double −/− mice have a smaller body shape, and most of them died between 3 and 4 weeks after birth, which may be due to the development of a symptom and disease similar to human acute myeloid leukemia (AML), a hematological malignant tumor that can cause a high mortality rate (Mullican et al., 2007). The AML-like symptoms caused by Nur77/NOR1 gene deficiency might be related to the increased expression of MYC, an oncogene whose expression is closely related to the pathogenesis of AML and which might be strongly suppressed by NOR1 in a Nur DNA-binding manner (Boudreaux et al., 2012). Bluteauet et al. had reported that NOR1 is a direct target of runt-related transcription factor 1 (RUNX1), one of the most important regulatory genes in hematopoiesis (Bluteau et al., 2011). Mutation and inactivation of RUNX1, which have been repeatedly observed in AML patients (Simon et al., 2020; Waidhauser et al., 2021), can induce proliferation of immature CD34^+^CD38^−^ progenitor cells and a concomitant decrease in NOR1 expression (Wang J. et al., 2021). Restoration of NOR1 expression can partially reduce the clonogenic potential of progenitor cells in AML patients (Simon et al., 2020). These results indicate that drugs that can enhance NOR1 expression or function may have therapeutic effects in AML. This hypothesis may be supported by recently published studies: 1) Z-ligustilide (Z-LIG), a major component of Ligusticum wallichii, was found to suppress the proliferation of human AML cells by promoting the recruitment of Ace-H3 (lys9/14) to the NOR1 promoter, thereby up-regulating NOR1 expression (Table 1) (Wang J. et al., 2021); and 2) SNDX-275, a histone deacetylase inhibitor, was found to promote pro-apoptotic signaling in leukemia cells in part by inducing NOR1 expression (Table 1) (Zhou et al., 2013).

NOR1 may also be a predictor of outcome in patients with diffuse large B-cell lymphoma (DLBCL): Researchers have observed a moderate increase in NOR1 levels in DLBCL patients who respond very well to chemotherapy, whereas a similar increase has been observed in patients who do not respond to chemotherapy (Shipp et al., 2002). Low NOR1 expression is also associated with poor clinical outcome in a cohort of 92 patients with DLBCL (Deutsch et al., 2017). It has been reported that the NR4A family member can be translocated from the nucleus to the mitochondria, where it has a pro-apoptotic effect (Thompson and Winoto, 2008; Thompson et al., 2010; Odagiu et al., 2021). Since NOR1 is a member of the NR4A family and the different types of NR4A family members have similar functions, it is possible that NOR1 enhances the apoptotic response to chemotherapy in curable DLBCL. This hypothesis can be supported by the following evidence: 1) genetic or pharmacological induction of NOR1 expression induces apoptosis in aggressive lymphoma cells and results in reduced tumor growth in a xenograft model, 2) NOR1 is positively correlated with pro-apoptotic genes in primary aggressive lymphomas, and 3) overexpression of NOR1 leads to strong induction of pro-apoptotic genes, including p53 upregulated modulator of apoptosis (PUMA), TNF related apoptosis inducing ligand (TRAIL), Bid, and Bik (Deutschet al., 2017). Similar to AML and DLBCL, where NOR1 gene abrogation contributes to the rapid postnatal development of AML in mice, it was also found that reduction of NOR1 expression was sufficient to induce mixed myelodysplastic/myeloproliferative neoplasms (MDS/MPNs) in mice (Ramirez-Herrick et al., 2011). The above results suggest that NOR1 mutation or loss of NOR1 function would promote the development of cancers.

Since NOR1 is a potential target for anticancer drug development, it is necessary to investigate the factors or drugs that regulate NOR1 expression or function. Indeed, this topic has been investigated in numerous studies. For example, researchers have found that the tumor suppressor p53 can increase the expression of PUMA and Bax (two pro-apoptotic genes) by up-regulating NOR1 expression, thereby helping to suppress tumor cell proliferation and promote tumor cell apoptosis (Fedorova et al., 2019). Dihydroergotamine, an ergot alkaloid used in the clinic to treat migraine, and alprostadil (ALP), also called prostaglandin E1 (PGE1), have been shown to induce NOR1 expression in AML cells by recruiting the superelongation complex to allow elongation of the NOR1 promoter, which pauses (Table 1) (Boudreaux et al., 2019). Prostaglandin A2 (PGA2) is also a trans-activating factor for NOR1 (Kagaya et al., 2005). It can directly bind the ligand-binding domain of NOR1, and the deficiency of this ligand-binding domain can inhibit the trans-activating effect of PGA2 on NOR1 (Table 1) (Kagaya et al., 2005). Considering that PGA2 is able to induce tumor cell apoptosis (Joubert et al., 2003), NOR1 may play an important role in the anti-tumor effect of PGA2. Because PGA2 is able to interact with NR4A2, researchers should also consider the possibility that PGA2 regulates tumor biology by interacting with NOR1, which should be investigated in future studies (Rajan et al., 2022).

Of particular note, in some recently published studies, nuclear expression of NOR1 is considered a sensitive and specific diagnostic marker for acinic cell carcinomas (ACCs), a low-grade malignant salivary gland tumor with cells showing serous acinar differentiation, because 1) NOR1 immunostaining was found in all ACC patients, whereas no similar immunostaining was found in non-ACC individuals (Jin et al., 2021; Owosho et al., 2021); and 2) NOR1 can distinguish from its mimics on fine-needle aspiration biopsy specimens (Nguyen et al., 2020). However, whether this positive expression of NOR1 is beneficial or detrimental to the progression of ACCs or the treatment and prognosis of ACC remains unclear. This question should be investigated in future studies. Given the similarities between the different NR4A types, investigators should also consider the role of NR4A1 (Nur77) and NR4A2 (Nurr1) in the pathogenesis of ACCs. Indeed, NR4A2 has emerged as a novel marker for ACC of the salivary glands lacking NOR1 increase (Haller et al., 2020).

## Conclusion

This review summarizes the function of NOR1 in various pathophysiological situations, such as the regulation of neuronal survival in brain development and the pathogenesis of hypoxic brain injury (Chio et al., 2016) and cerebral ischemia (Kim, et al., 2006), the regulation of fear memory formation (Volpicelli et al., 2007; Hawk et al., 2012; Bridi et al., 2017), the regulation of cardiovascular disorders associated with atherosclerosis and cardiac hypertrophy, such as monocyte-endothelial cell adhesion (Martorell et al., 2007), endothelial cell growth (Martorell et al., 2008), cardiomyocyte hypertrophy (Myers et al., 2009; Feng et al., 2015; Cañes et al., 2020), and VSMC proliferation (Martínez-González et al., 2003; Bi et al., 2013; Alonso et al., 2018). NOR1 can also regulate the inflammatory response (Pei et al., 2005; Bonta et al., 2006; McMorrow and Murphy, 2011; De Paoli et al., 2015; Rodríguez-Calvo et al., 2017; Hafiane and Daskalopoulou, 2022) and induce thymocyte apoptosis by re-localizing mitochondria (Thompson et al., 2010). NOR1 is also considered a potential drug target for the treatment of liver fibrosis (Vacca et al., 2013), metabolic disorders associated with an imbalance of lipogenesis and glucose homeostasis (Pei et al., 2006; Myers et al., 2009; Oita et al., 2009), and osteoarthritis (Ma et al., 2020) or rheumatoid arthritis (Andreas et al., 2009). In tumor pathogenesis, NOR1 shows a negative correlation with tumor progression, and enhanced NOR signaling is able to suppress tumor cell growth (Shipp et al., 2002; Mullican et al., 2007; Ramirez-Herrick et al., 2011; Boudreaux et al., 2012; Deutsch et al., 2017; Zhang et al., 2018; Boudreaux et al., 2019; Fedorova et al., 2019; Haller et al., 2019; Son et al., 2019; Wang et al., 2020; Safe and Karki, 2021). Based on the above findings, we summarize the drugs and small molecules that regulate NOR1 expression and function (Table 1). Future studies need to identify endogenous ligands for NOR1 to further deepen our understandings of the role of NOR1 in the pathogenesis of various types of diseases.
